# Natural astaxanthin enhanced antioxidant capacity and improved semen quality through the MAPK/Nrf2 pathway in aging layer breeder roosters

**DOI:** 10.1186/s40104-021-00633-8

**Published:** 2021-11-04

**Authors:** Shan Gao, Nuo Heng, Fang Liu, Yong Guo, Yu Chen, Liang Wang, Hemin Ni, Xihui Sheng, Xiangguo Wang, Kai Xing, Longfei Xiao, Xiaolong Qi

**Affiliations:** 1 Animal Science and Technology College, Beijing University of Agriculture, Beijing, 102206 China; 2 School of Economics and Management, Beijing University of Agriculture, Beijing, 102206 China; 3 Department of Livestock and Poultry Products Testing, Beijing General Station of Animal Husbandry, Beijing, 100107 China

**Keywords:** Aging rooster, Antioxidant capacity, MAPK/Nrf2 pathway, Natural astaxanthin, Semen quality

## Abstract

**Background:**

Natural astaxanthin (ASTA) has strong antioxidant properties and has been widely used as a health product to improve human health. However, the effects of ASTA on the reproductive performance of aging roosters have been poorly studied. We aimed to investigate the effects of dietary ASTA on semen quality and antioxidant capacity in aging roosters and to explore the potential mechanism of semen quality change via anti-oxidation defense system.

**Methods:**

In the present study, 96 53-week-old Jinghong No. 1 layer breeder roosters were fed a corn-soybean meal basal diet containing 0, 25, 50, or 100 mg/kg ASTA for 6 weeks.

**Results:**

Semen quality in the ASTA groups remarkably improved than that in the control group, and antioxidant activities, the abilities to scavenge hydroxyl radicals and superoxide anions, increased gradually with ASTA addition (*P* < 0.05). In addition, the mRNA levels of antioxidant enzymes as well as the mRNA and protein levels of the mitogen-activated protein kinase (MAPK) and nuclear factor-erythroid 2-related factor 2 (Nrf2) were markedly increased in the 50–100 mg/kg ASTA group (*P* < 0.05).

**Conclusions:**

Collectively, these results demonstrate that dietary ASTA may improve semen quality by increasing antioxidant enzyme activities and the ability to scavenge hydroxyl radicals, which may be related to upregulation of the MAPK/Nrf2 pathway.

## Introduction

In poultry production, assisted reproductive technology such as artificial insemination is widely used. A single male is responsible for fertilizing dozens of female birds [1]. Therefore, the reproductive performance of roosters is a key factor in production. However, the sperm quality of roosters decreases gradually at approximately 50–55 weeks [2]. Several important reproductive traits change in aging roosters, including decreases in the sperm concentration, motility, testosterone level, and antioxidant capacity [3, 4]. Male fertility is one of the most important factors in determining hatchability. Therefore, it is necessary to find a way to alleviate the influence of age on the reproductive characteristics of breeding roosters. Recently, studies have shown that dietary supplementation with natural anti-oxidative compounds such as dietary linseed oil [5], lycopene [6], and curcumin [7] can alleviate the negative effects of age on roosters.

Astaxanthin (3,3-dihydroxy-β, β-carotene-4,4-dione) is a xanthophyll carotenoid, that is mainly divided into two forms: natural astaxanthin (ASTA) and synthetic astaxanthin. ASTA can be extracted from algae [8], yeast [9], fish, krill, and some microorganisms [10]. The *Haematococcus pluvialis*, a green microalga, is one of the major sources of ASTA, and a large amount of ASTA can be extracted from it under stress conditions [11, 12]. Although synthetic astaxanthin has the same chemical formula as ASTA, the ability to scavenge singlet oxygen and free radical of ASTA is over 50 times and 20 times that of synthetic astaxanthin, respectively [13]. Moreover, the European Commission considers ASTA as a food dye [14]. Currently, ASTA has received much attention for its various beneficial characteristics, including its antioxidant [15], anti-inflammatory [16], and anti-apoptotic properties [17]. However, there is little information on whether ASTA can improve the male reproductive performance.

The mitogen-activated protein kinase (MAPK) pathway, a family of serine/threonine protein kinases, is widely conserved in eukaryotes and is involved in antioxidant enzyme expression by modulating the Nrf2/ARE axis [18, 19]. Nuclear factor-erythroid 2-related factor 2 (Nrf2) is an important regulator of antioxidant responsive element (ARE)-activated gene expression. Nrf2 can repair and degrade of damaged macromolecules caused by oxidative stress by upregulating the expression of antioxidant enzyme genes [20]. Studies have shown that the MAPK/Nrf2 pathway improves the antioxidant capacity of eggs by upregulating the mRNA expression of *p38MAPK*, *Nrf2*, and *HO-1* [21]. Moreover, the Nrf2/ARE axis alleviates meat lipid peroxidation by regulating the expression of antioxidant enzymes and phase II enzymes to achieve long-term meat storage [22]. However, the potential effect of ASTA on sperm quality, antioxidant capacity, and the MAPK/Nrf2 antioxidant pathway in aging layer roosters was not clear.

Therefore, in the current study, we investigated our hypothesis that ASTA can improve semen quality by enhancing antioxidant capacity and activating the MAPK/Nrf2 pathway in aging roosters.

## Materials and methods

### Animal care and use

All experimental protocols were approved by the Animal Care and Use Committee of the Beijing University of Agriculture.

### Experimental design

In the present study, a total of ninety-six 53-week old Jinghong No.1layer breeder roosters with a similar weight and genetic background were supplied by Beijing Huadu Group Co., Ltd. (Beijing, China), and the birds were randomly distributed into four treatment groups, following a completely randomized design. Each treatment had six replicates, and there were four birds per replicate with one bird per cage (70 cm × 60 cm × 75 cm). All birds were fed a basal diet for 1 week and then assigned to a corn-soybean meal-based diet containing 0, 25, 50, and 100 mg/kg ASTA for 6 weeks. *Haematococcus pluvialis* was purchased from Jingzhou Natural Astaxanthin Inc. (Jingzhou, China), and the ASTA content was 1.54%. The composition and nutrient levels of the corn soybean meal-based diet are shown in Table 1. During the experimental period, the birds had 16-h light cycles and were allowed ad libitum access to water and the treatment diets.

**Table 1:** Composition and nutrient content of basal diets (air dried basis, %)

Ingredients	Content, %	Nutrition level	
Corn	69.93	Metabolizable energy, MJ·kg^−1^	12.02
Soybean meal	18.60	Crude protein, %	14.00
Wheat bran	3.80	Methionine, %	0.23
Soybean oil	1.40	Lysine, %	0.64
Limestone	2.60	Calcium, %	1.37
Dicalcium phosphate	1.80	Total phosphorus, %	0.63
Salt	0.25	Available phosphorus, %	0.44
Choline chloride	0.20		
Premix^1^	1.42		
Total	100.00		

^1^The premix provided the following per kilogram of the diet: Cu 10 mg, Fe 80 mg, Mn 100 mg, Zn 80 mg, VA 20,000 IU, VD 3,000 IU, VE 30 IU, VK 2 mg, VB_1_ 2 mg, VB_2_ 10 mg, VB_6_ 6 mg, VB_12_ 0.012 mg, folic acid 1.2 mg, D-pantothenic acid 12 mg, nicotinic acid 40 mg, biotin 0.2 mg, and Se 0.3 mg

### Semen collection and analysis

Birds were trained for semen collection every 2 weeks. Semen samples were randomly collected from each bird in each replicate once every two weeks using the abdominal massage method [23]. Next, semen volume was measured with a graduated collecting tube. Semen samples were diluted in a pre-warmed (37 °C) saline solution (0.9%, 1:49), after which samples were assessed for seminal characteristics using CASA system (WLJX-9000 Weili Color Sperm Analysis System; Weili New Century Science & Tech Dev., Beijing, China), including semen concentration and sperm motility parameters such as sperm motility (%), average path velocity (VAP, μm/s), straight linear velocity (VSL, μm/s), curvilinear velocity (VCL, μm/s), amplitude of lateral head displacement (ALH, μm), straightness (STR, %), linearity (LIN, %), wobble of the curvilinear trajectory (WOB, %) and beat cross frequency (BCF, Hz) [24].

Sperm viability was evaluated using the eosin-nigrosin staining method [25]. Briefly, a 5 μL sperm sample was mixed with 25 μL of the stain on a pre-warmed slide before the test. Then, the stain was spread with another slide to make a sperm suspension smear. An Olympus BX51 microscope (Olympus Corporation, Tokyo, Japan) was used at a final magnification of 400 to analyze the sperm viability. For each sperm sample, five microscopic fields were observed subjectively, and 200 sperms were counted to evaluate viability. Sperm with unstained heads of spermatozoa were considered viable, while sperm displaying partial or complete purple staining were considered nonviable.

### Antioxidant enzyme and oxygen free radical analyses

After analyzing the semen quality, the semen sample was centrifuged at 4,000 × *g* for 10 min at 4 °C, and then the seminal plasma was separated and stored at − 20 °C. At the end of the 6-week feeding trial, one bird from each replicate was randomly selected and sacrificed by cervical dislocation [5]. Immediately after euthanasia, blood samples were collected from the left jugular vein of the birds. Plasma was separated by centrifugation at 3,000 × *g* for 10 min and stored at − 20 °C until analysis. The testes, liver, and kidneys were removed immediately and quickly frozen at − 80 °C for subsequent analysis. These samples were used to analyze the activities of antioxidant enzymes and the ability to scavenge oxygen free radicals. Briefly, antioxidant capacity included the glutathione peroxidase (GSH-Px), superoxide dismutase (SOD), catalase (CAT), and total antioxidant capacity (T-AOC), while oxygen free radical scavenging abilities included hydroxyl radical scavenging ability and superoxide anion scavenging ability. Moreover, malondialdehyde (MDA), as the product of lipid metabolism, was analyzed using a spectrophotometric method. These assays were performed using a commercial kit (Nanjing Jiancheng Bio-Engineering Institute, Nanjing, China). The method and principle used to determine the antioxidant indicators with these kits were previously described elsewhere [26].

### Quantitative PCR analysis

The mRNA expression levels of *p38*, extracellular signal-regulated kinase (*ERK*), c-Jun N-terminal kinase 1 (*JNK1*), c-Jun N-terminal kinase 2 (*JNK2*), c-Jun N-terminal kinase 3 (*JNK3*), *Nrf2*, Cu-Zn superoxide dismutase (*SOD1*), Mn superoxide dismutase (*SOD2*), *CAT*, glutathione peroxidase 1 (*GPX1*), and peroxidase 4 (*GPX4*) were determined via real-time quantitative PCR (RT-qPCR) with β-actin (*ACTB*) as an internal control in the testis tissues. Total RNA was isolated from 0.1 g of the testis sample using TRIzol reagent (Invitrogen, Carlsbad, CA, USA) according to the manufacturer’s protocol. Then, 1% agarose gel stained with ethidium bromide was used to confirm the presence of 18S and 28S bands. Finally, the total RNA concentration was measured using a spectrophotometer at 260 nm. Total RNA was reverse transcribed to cDNA using a Thermo Fisher First cDNA Synthesis Kit (#K1621; Thermo Fisher Scientific, Waltham, MA, USA). A Step One Plus Real-time PCR system (Applied Biosystems, Foster City, CA, USA) was used for the quantitative PCR analyses. The primers for the selected genes are listed in Table 2. After initial denaturation at 95 °C for 10 min, 40 cycles of amplification were performed (95 °C for 10 s and 58 °C for 30 s), generating melt curves to verify amplification specificity. The relative gene expression levels were calculated using the 2^−ΔΔCt^ method [27], with *ACTB* as the reference gene.

**Table 2:** Primers used for the real-time quantitative PCR

Genes	Primer sequence (5′ → 3′)	Fragment length, bp	Annealing temperature, °C	Accession number
*ACTB*	Forward: GCCAACAGAGAGAAGATGACAC	118	58	NM_205518
	Reverse: GTAACACCATCACCAGAGTCCA			
*SOD1*	Forward: TTGTCTGATGGAGATCATGGCTTC	98	58	NM_205064
	Reverse: TGCTTGCCTTCAGGATTAAAGTGAG			
*SOD2*	Forward: CAGATAGCAGCCTGTGCAAATCA	86	58	NM_204211.1
	Reverse: GCATGTTCCCATACATCGATTCC			
*CAT*	Forward: ACCAAGTACTGCAAGGCGAAAGT	91	58	NM_001031215.2
	Reverse: ACCCAGATTCTCCAGCAACAGTG			
*GPX-1*	Forward: TTCGAGAAGTTCCTCGTGGG	79	58	NM_0012778553.2
	Reverse: CCTGCAGTTTGATGGTCTCG			
*GPX-4*	Forward: TCAACCGTGAGGGCCAAGT	100	58	NM_001346448.1
	Reverse: CTCGGCACGCAGCTCTAC			
*Nrf2*	Forward: ACATGGACAGTTCTCCTGGG	92	58	NM_205117.1
	Reverse: CGGCTCCACAGAAGGAAGTA			
*ERK*	Forward: AGCAAGCTTTAGCCCATCCA	108	58	NM_204150.1
	Reverse: CCTTCGGCAAGTCATCCAAT			
*JNK1*	Forward: GGGTGCATTATGGGCGAAAT	108	58	XM_421650.2
	Reverse: TTCTGGGCACGGTGTTCCTA			
*JNK2*	Forward: AGCAGCCTCGATGCCTTGAC	110	58	AB000807.1
	Reverse: CAAGCAATTCAGGCCCAATG			
*JNK3*	Forward: CTGGTGAGTGAGCTGATGGA	82	58	NM_001318224.3
	Reverse: ACAGCAGGTAGGACATTCGT			
*p38*	Forward: TGTGTTCACCCCTGCCAAGT	149	58	AJ719744.1
	Reverse: GCCCCCGAAGAATCTGGTAT			

### Western blotting analysis

The protein expression of Nrf2, p38, p-p38, ERK, p-ERK, JNK, and p-JNK was evaluated by western blotting in the testis tissues, and the antibodies used are shown in Table 3. Total proteins were extracted from testis tissues using RIPA Buffer (W1001; SinoGene, Beijing, China) according to the manufacturer’s instructions. The protein concentration of the extracts was determined using the Bradford method (W1014; SinoGene). Samples containing 30 μg of protein and protein markers (SM26616; Thermo Fisher Scientific) were separated by sodium dodecyl sulfate polyacrylamide gel electrophoresis (SDS-PAGE; 4% stacking gel, 12% separating gel) at 120 V for 2 h. Proteins were transferred to polyvinylidene difluoride (PVDF) membranes using a Bio-Rad mini transfer system (Bio-Rad Laboratories, Hercules, CA, USA). The membranes were blocked with Fast Protein-free Block Buffer (#W1028; SinoGene) for 5 min and then incubated with primary antibody (1:1,000 dilution) overnight at 4 °C. After rinsing four times with Tris-buffered saline with 0.1% Tween^®^ 20 Detergent (TBST) for 10 min per rinse, HRP-conjugated secondary antibody (SA00001–2, ptg) was applied to the membranes at a dilution of 1:3,000 for 1 h. Then, after rinsing three times with TBST for 10 min per rinse, immunological signals were detected using a chemiluminescent (ECL) kit (29,050; Engreen, Biosystem, Ltd.) and exposed to X-ray films in the dark. Protein bands were quantified by densitometric analysis using Image J analysis software (National Institutes of Health, Bethesda, MD, USA).

**Table 3:** Antibodies used for the western blot analysis

Antibodies	Cat NO.	Source	Dilution
p38	9212	Cell Signaling Technology, Danvers, MA, USA	1:1,000
p-p38	4511	Cell Signaling Technology, Danvers, MA, USA	1:1,000
ERK	4695	Cell Signaling Technology, Danvers, MA, USA	1:1,000
p-ERK	9101	Cell Signaling Technology, Danvers, MA, USA	1:1,000
JNK	9252	Cell Signaling Technology, Danvers, MA, USA	1:1,000
p-JNK	4668	Cell Signaling Technology, Danvers, MA, USA	1:1,000
Nrf2	ab31163	Abcam, Cambridge, UK	1:5,000
β-Actin	AC028	Abclonal, Woburn, MA, USA	1:3,000

### Statistical analysis

Statistical analysis was performed using SPSS 22.0 (IBM Corp., Armonk, NY, USA). Data related to the effect of dietary ASTA levels on semen quality, antioxidant capacity, and gene and protein expression were analyzed by one-way analysis of variance with orthogonal linear and quadratic contrasts. Duncan’s multiple comparison test was used to examine statistical differences among the treatments. Statistical significance was defined as *P* < 0.05.

## Results and discussion

### Semen quality

Semen quality is a crucial factor in roosters’ fertility because the fertility rate is positively correlated with sperm motility, sperm concentration, and sperm motility parameters in roosters [28]. Few reports have described the effects of ASTA on semen quality in aging roosters. We evaluated the effects of dietary ASTA on semen quality in aging roosters (Tables 4, 5**)**. Briefly, there were no differences in semen quality between the control group and the ASTA groups before the commencement of the treatment (preparation period). The semen volume was significantly higher in the 25 mg/kg ASTA group than that in the other groups at week 6 (*P* < 0.05). Moreover, with an increase in dietary ASTA, sperm viability and sperm concentration significantly increased compared to those in the control group (*P* < 0.05). In particular, the sperm concentration was linearly and quadratically affected (*P* < 0.05) by the dietary ASTA levels. In addition, the sperm motility parameters of aging layer roosters in the 50 mg/kg ASTA group were significantly linearly increased relative to those in the control group (*P* < 0.05). Moreover, sperm motility increased from 64.40% to 76.23% in the 0–100 mg/kg ASTA-treated group (*P* < 0.05). It has been previously demonstrated that sperm viability, total motility, and sperm kinematic parameters increase after ASTA treatment in mice [29]. A recent study also showed similar results that ASTA can improve post-thawed rooster sperm motility and kinetic parameters, including total motility, progressive motility, VAP, VSL, and LIN. However, there was no significant difference in LIN, STR, ALH, and BCF in the ASTA treatment groups compared to those in the control [30]. Interestingly, the opposite result was observed in the current study that the motility parameters including STR, LIN, WOB, VSL, VAP, BCF, and ALH in the ASTA groups were significantly higher than those in the control group (*P* < 0.05). The discrepant results may be partly related to the concentration of ASTA added, the duration of supplementation, and the test subjects. Taken together, these findings indicate that dietary ASTA supplementation significantly increased the semen quality during the test period (0–6 weeks). However, the possible mechanisms by which dietary ASTA affects semen quality are not well understood, but may be related to the antioxidant defense system.

**Table 4:** Effect of dietary natural astaxanthin (ASTA) supplementation on the semen volume, sperm viability, and sperm concentration of aging layer breeder roosters

Items	Time, week	ASTA levels, mg/kg				SEM		*P*-value	
		0	25	50	100		ANOVA	Linear	Quadratic
Semen volume, mL	0	0.25	0.26	0.26	0.24	0.005	0.606	0.769	0.199
	2	0.25^b^	0.31^a^	0.27^b^	0.27^b^	0.006	< 0.01	0.429	< 0.01
	4	0.24^b^	0.31^a^	0.29^a^	0.29^a^	0.006	< 0.01	< 0.01	< 0.01
	6	0.25^b^	0.34^a^	0.32^a^	0.31^a^	0.054	< 0.01	< 0.01	< 0.01
Sperm viability, %	0	83.83	81.33	82.17	82.83	0.420	0.185	0.549	0.060
	2	85.33^b^	89.17^a^	90.83^a^	90.83^a^	0.650	< 0.01	< 0.01	0.063
	4	83.17^c^	88.67^b^	90.17^ab^	91.50^a^	0.750	< 0.01	< 0.01	0.011
	6	85.67^c^	91.33^ab^	91.00^b^	94.67^a^	0.870	< 0.01	< 0.01	0.400
Sperm concentration, 10^8^/mL	0	11.36	11.28	11.30	11.37	3.150	0.742	0.862	0.291
	2	11.37^b^	11.49^b^	11.84^a^	11.96^a^	5.427	< 0.01	< 0.01	0.931
	4	11.37^c^	12.04^b^	12.15^b^	12.42^a^	8.489	< 0.01	< 0.01	< 0.01
	6	11.33^c^	12.57^b^	12.62^b^	12.97^a^	13.523	< 0.01	< 0.01	< 0.01

^a–c^ Means within a row with no common superscripts differ significantly (*P* < 0.05). Data represent the mean of six replicates. Abbreviations: *SEM*, standard error of the mean; *ANOVA*, analysis of variance

**Table 5:** Effect of dietary natural astaxanthin (ASTA) supplementation on the sperm motility parameters of aging layer breeder roosters

Items	Time, week	ASTA levels, mg/kg				SEM		*P*-value	
		0	25	50	100		ANOVA	Linear	Quadratic
Sperm motility, %	0	63.07	61.52	63.00	61.95	0.502	0.649	0.687	0.811
	2	62.55^b^	65.20^ab^	67.95^a^	66.51^a^	0.615	< 0.01	< 0.01	0.049
	4	62.00^c^	68.83^b^	69.78^ab^	71.67^a^	0.851	< 0.01	< 0.01	< 0.01
	6	64.40^b^	65.77^b^	73.41^a^	76.23^a^	1.229	< 0.01	< 0.01	0.611
STR, %	0	79.76	79.73	79.51	79.32	0.783	0.997	0.839	0.965
	2	78.08^c^	84.22^b^	87.14^a^	84.77^ab^	0.815	< 0.01	< 0.01	< 0.01
	4	79.24^c^	84.27^b^	87.38^a^	86.64^a^	0.747	< 0.01	< 0.01	< 0.01
	6	76.53^b^	85.28^a^	86.74^a^	87.41^a^	1.013	< 0.01	< 0.01	< 0.01
LIN, %	0	44.78	44.57	45.51	43.03	0.429	0.224	0.259	0.186
	2	45.16^c^	53.48^b^	55.59^a^	55.96^a^	0.990	< 0.01	< 0.01	< 0.01
	4	48.07^c^	54.10^b^	57.38^a^	57.47^a^	0.857	< 0.01	< 0.01	< 0.01
	6	45.47^c^	61.14^a^	61.08^a^	58.09^b^	1.430	< 0.01	< 0.01	< 0.01
WOB, %	0	52.73	53.16	53.07	52.09	0.408	0.811	0.604	0.422
	2	53.90^c^	58.39^b^	59.62^b^	63.09^a^	0.787	< 0.01	< 0.01	0.542
	4	54.27^c^	61.10^ab^	59.13^b^	63.07^a^	0.774	< 0.01	< 0.01	0.080
	6	52.96^c^	69.57^a^	71.07^a^	64.15^b^	1.620	< 0.01	< 0.01	< 0.01
VCL, μm/s	0	63.23	61.61	62.29	62.57	0.396	0.572	0.726	0.256
	2	62.40^c^	67.73^b^	76.29^a^	69.39^b^	1.099	< 0.01	< 0.01	< 0.01
	4	60.63^d^	70.74^c^	80.97^b^	85.91^a^	2.142	< 0.01	< 0.01	0.096
	6	59.89^c^	73.60^b^	80.76^a^	83.07^a^	2.009	< 0.01	< 0.01	< 0.01
VSL, μm/s	0	29.60	29.75	29.38	28.56	0.447	0.810	0.416	0.607
	2	31.67^c^	32.72^bc^	36.37^b^	46.35^a^	1.354	< 0.01	< 0.01	< 0.01
	4	30.82^c^	38.92^b^	41.51^b^	45.98^a^	1.268	< 0.01	< 0.01	0.132
	6	30.02^b^	40.53^a^	37.13^a^	41.16^a^	1.198	< 0.01	< 0.01	0.072
VAP, μm/s	0	32.26	32.00	31.06	32.38	0.406	0.834	0.939	0.458
	2	31.19^d^	35.27^c^	41.78^b^	47.13^a^	1.332	< 0.01	< 0.01	0.468
	4	33.00^c^	44.31^b^	45.59^b^	53.38^a^	1.629	< 0.01	< 0.01	0.148
	6	32.09^c^	46.59^a^	47.37^a^	41.52^b^	1.439	< 0.01	< 0.01	< 0.01
BCF, Hz	0	3.57	3.53	3.67	3.55	0.032	0.423	0.802	0.498
	2	3.53^c^	3.81^b^	3.97^a^	4.05^a^	0.045	< 0.01	< 0.01	0.024
	4	3.53^c^	4.07^b^	4.60^a^	4.14^b^	0.084	< 0.01	< 0.01	< 0.01
	6	3.59^c^	3.89^b^	4.44^a^	4.38^a^	0.079	< 0.01	< 0.01	< 0.01
ALH, μm	0	2.20	2.28	2.18	2.20	0.145	0.645	0.776	0.602
	2	2.12^c^	2.31^bc^	2.46^b^	2.96^a^	0.076	< 0.01	< 0.01	0.082
	4	2.35^b^	3.10^a^	3.05^a^	3.30^a^	0.087	< 0.01	< 0.01	0.015
	6	2.11^c^	3.00^b^	3.02^b^	3.36^a^	0.107	< 0.01	0.011	< 0.01

^a–d^ Means within a row with no common superscripts differ significantly (*P* < 0.05). Data represent the mean of six replicates. Abbreviations: *SEM*, standard error of the mean; *ANOVA*, analysis of variance; *STR*, straightness; *LIN*, linearity; *WOB*, wobble of the curvilinear trajectory; *VCL*, curvilinear velocity; *VSL*, straight linear velocity; *VAP*, average path velocity; *BCF*, beat cross frequency; *ALH*, amplitude of lateral head displacement

### Antioxidant enzyme activity and free radical scavenging ability

**Fig. 1:**
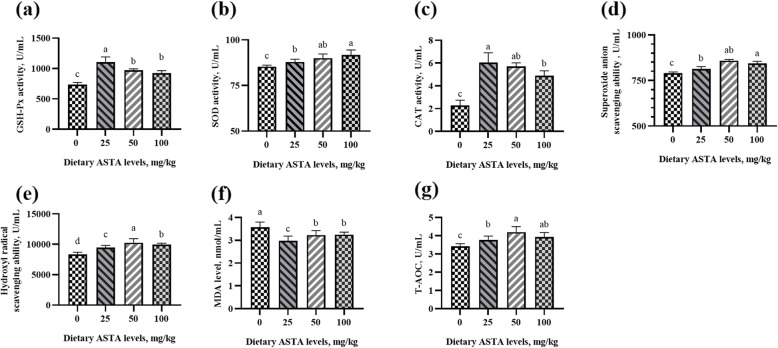
Effect of dietary natural astaxanthin (ASTA) supplementation on the antioxidant enzyme activity and free radicals in the plasma of aging layer breeder roosters. **(a-c)** Glutathione peroxidase (GSH-Px), superoxide dismutase (SOD), and catalase (CAT) activity in the plasma. **(d-e)** Scavenging free radical abilities in the plasma. **(f)** Malondialdehyde (MDA) level in the plasma. **(g)** Total antioxidant capacity (T-AOC) in the plasma. The data represent mean ± standard deviation; *n* = 6 in each group. ^a–d^ Means within a row with no common superscripts differ significantly (*P* < 0.05)

**Fig. 2:**
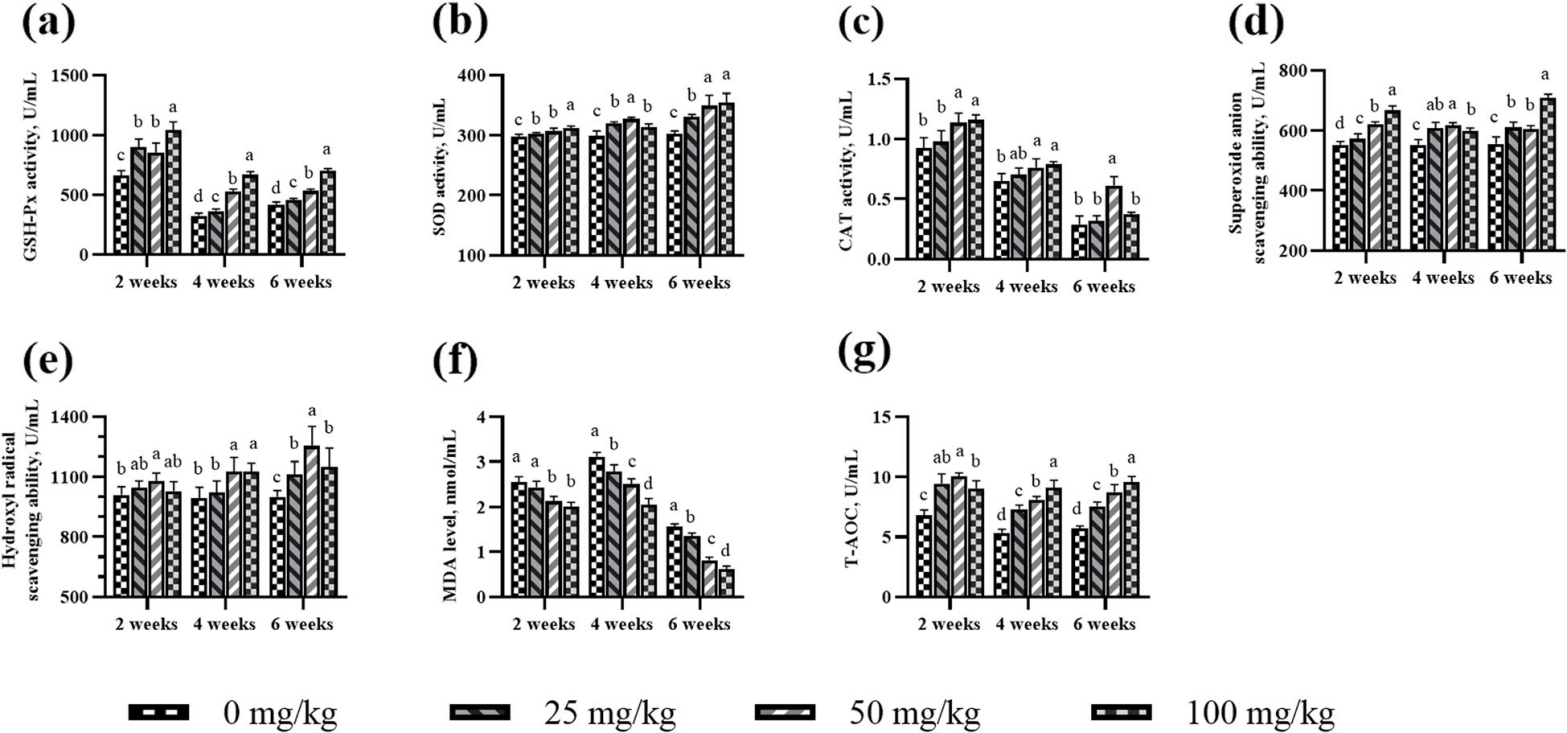
Effect of dietary natural astaxanthin (ASTA) supplementation on antioxidant enzyme activity and free radicals in the seminal plasma of aging layer breeder roosters. **(a-c)** Glutathione peroxidase (GSH-Px), superoxide dismutase (SOD), and catalase (CAT) activity in the seminal plasma. **(d-e)** Scavenging free radical abilities in the seminal plasma. **(f)** Malondialdehyde (MDA) level in the seminal plasma. **(g)** Total antioxidant capacity (T-AOC) in the seminal plasma. The data represent mean ± standard deviation; *n* = 6 in each group. ^a–d^ Means within a row with no common superscripts differ significantly (*P* < 0.05)

**Fig. 3:**
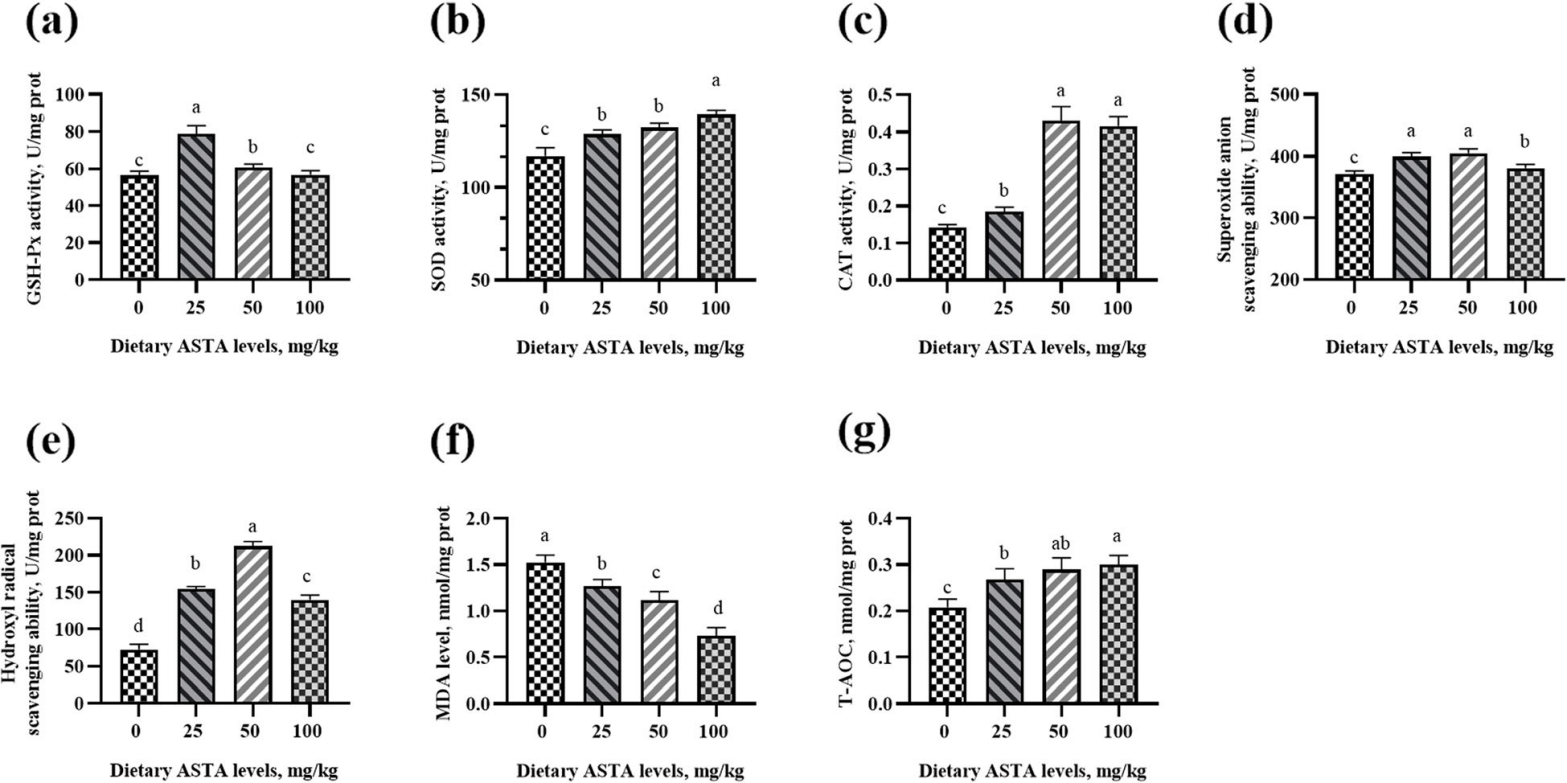
Effect of dietary natural astaxanthin (ASTA) supplementation on the antioxidant enzyme activity and free radicals in the testes of aging layer breeder roosters. **(a-c)** Glutathione peroxidase (GSH-Px), superoxide dismutase (SOD), and catalase (CAT) activity in the testes. **(d-e)** Scavenging free radical abilities in the testes. **(f)** Malondialdehyde (MDA) level in the testes. **(g)** Total antioxidant capacity (T-AOC) in the testes. The data represent mean ± standard deviation; *n* = 6 in each group. ^a–d^ Means within a row with no common superscripts differ significantly (*P* < 0.05)

**Fig. 4:**
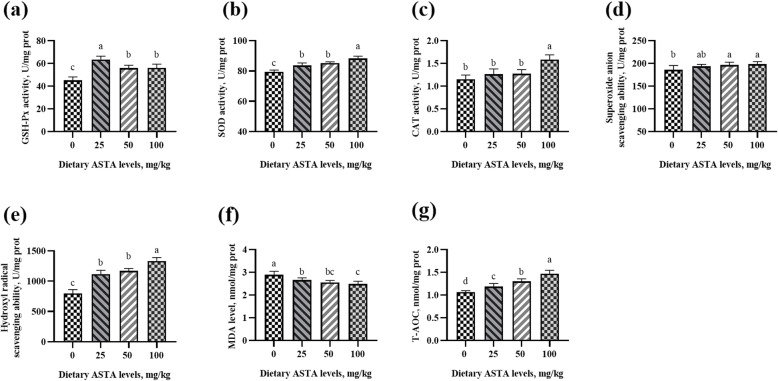
Effect of dietary natural astaxanthin (ASTA) supplementation on the antioxidant enzyme activity and free radicals in the liver of aging layer breeder roosters. **(a-c)** Glutathione peroxidase (GSH-Px), superoxide dismutase (SOD), and catalase (CAT) activity in the liver. **(d-e)** Scavenging free radical abilities in the liver. **(f)** Malondialdehyde (MDA) level in the liver. **(g)** Total antioxidant capacity (T-AOC) in the liver. The data represent mean ± standard deviation; *n* = 6 in each group. ^a–d^ Means within a row with no common superscripts differ significantly (*P* < 0.05)

**Fig. 5:**
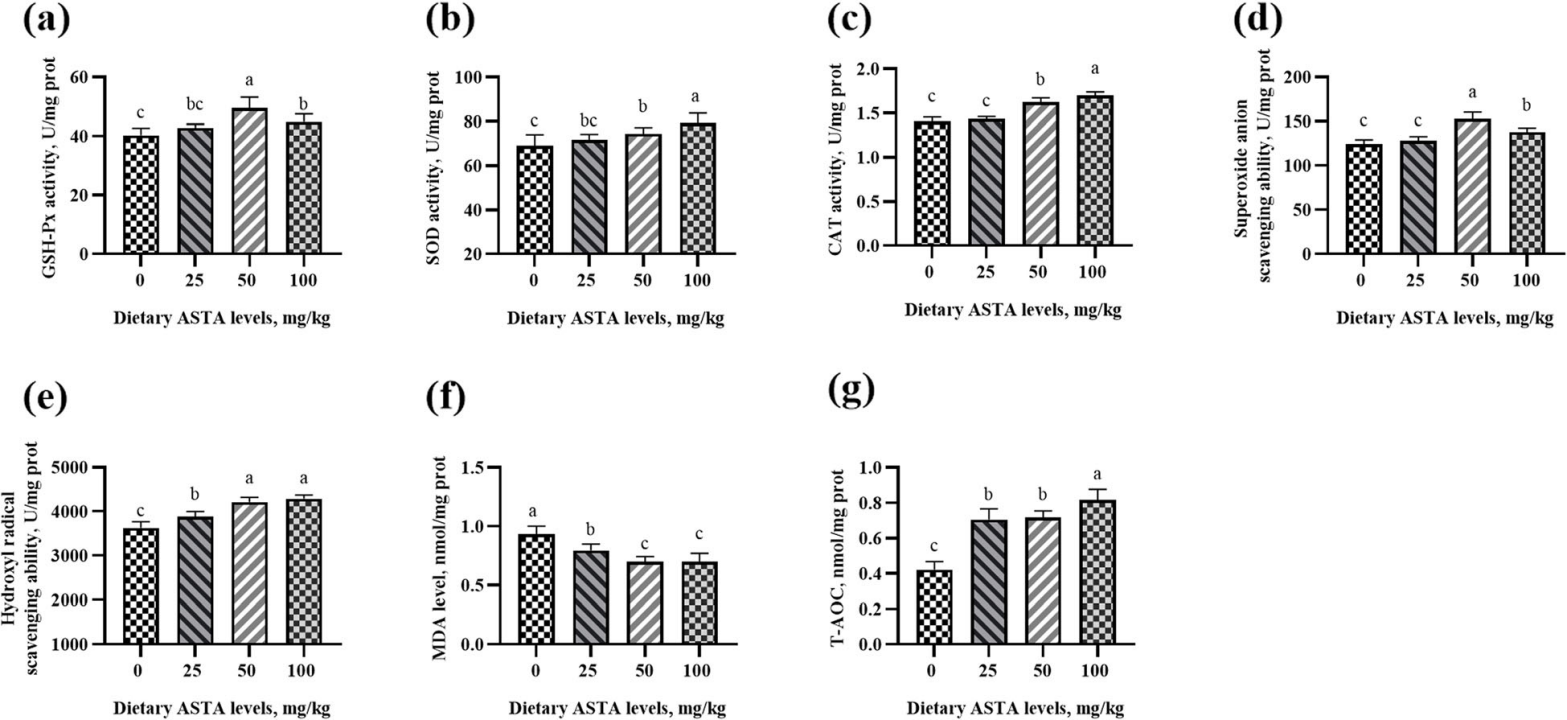
Effect of dietary natural astaxanthin (ASTA) supplementation on the antioxidant enzyme activity and free radicals in kidneys of aging layer breeder roosters. **(a-c)** Glutathione peroxidase (GSH-Px), superoxide dismutase (SOD), and catalase (CAT) activity in the kidney. **(d-e)** Scavenging free radicals’ abilities in the kidneys. **(f)** Malondialdehyde (MDA) level in the kidneys. **(g)** Total antioxidant capacity (T-AOC) in the kidneys. The data represent mean ± standard deviation; *n* = 6 in each group. ^a–c^ Means within a row with no common superscripts differ significantly (*P* < 0.05)

In aging roosters, a decrease in the antioxidant defense system, makes rooster spermatozoa vulnerable to lipid peroxidation [31]. Other studies have reported that senescence occurs due to the damage caused by free radicals and reactive oxygen species (ROS) to DNA, lipids, and proteins [32, 33]. Sophisticated enzymatic (SOD, CAT and GSH-Px) and non-enzymatic (vitamins A, C, E, and carotenoids) antioxidants constitute an antioxidant defense system that can regulate overall ROS levels to maintain physiological homeostasis [34]. ASTA, as a natural carotenoid antioxidant, can be transferred to the right place in the tissues and exert antioxidant effects at an appropriate concentration [35]. Previous studies haves demonstrated that ASTA has a higher antioxidant activity relative to those of α-carotene, lycopene, lutein, and β-carotene [36], and the capacity of scavenging singlet oxygen is approximately 550 times more than that of vitamin E [37]. The effects of dietary ASTA addition on antioxidant enzyme activity and free radical scavenging ability in the plasma, seminal plasma, testes, liver, and kidneys of aging layer breeder roosters in the present study are shown in Figs. 1, 2, 3, 4 and 5. Briefly, the 25 mg/kg ASTA treatment linearly and quadratically increased the GSH-Px activity in the plasma, testes, and liver of aging layer roosters compared with that in the control group (*P* < 0.05). The activities of SOD and CAT also increased gradually with increasing dietary ASTA supplementation from 50 to 100 mg/kg (*P* < 0.05). Furthermore, MDA concentrations decreased linearly with increasing dietary ASTA levels (*P* < 0.05) in the birds. With an increase in dietary ASTA, the levels of T-AOC increased linearly (*P* < 0.05). This observation is consistent with a previous report that in general, the activities of SOD, CAT, and GSH-Px in the brain and liver of rats increased dramatically at treatment with ASTA [38, 39]. In addition, we found that dietary ASTA supplementation linearly and quadratically increased the abilities of scavenging hydroxyl radicals and superoxide anions compared with those in the control group, which was consistent with a previous study [40]. In particular, the abilities of scavenging hydroxyl radical and superoxide anion of aging roosters were significantly increased in the 50 mg/kg ASTA group (*P* < 0.05).

### Relationship between semen quality and antioxidant enzyme activity

The semen volume, sperm concentration, viability, and forward motility decrease, whereas lipid peroxidation in seminal plasma increases in male poultry over 45 weeks of age [41]. Therefore, we aimed to improve the quality of semen by improving the antioxidant capacity of aging roosters using dietary ASTA in the present study. Moreover, it has been demonstrated that antioxidant capacity plays an important role in maintaining sperm viability because it can protect against lipid peroxidation in semen [42]. MDA is an important indicator of lipid peroxidation. A previous study showed that sperm motility was negatively correlated with MDA content in spermatozoa [43]. In the current study, the MDA content in the semen plasma, plasma, and tissues showed a dose-dependent decrease with changes in dietary ASTA. Similar findings were obtained in rats in which ASTA treatment group showed a significant increase in SOD and CAT activities, a significant decrease in MDA levels, as well as repaired oxidative stress-induced fertility disorder [44]. Therefore, the improvement of semen quality in aging roosters may be related to two factors. First, antioxidant enzyme activity remarkably improved, and MDA concentration decreased linearly with increasing dietary ASTA concentration, which plays an important role in the prevention of oxidative damage in the sperm plasma membrane. Second, dietary ASTA may improve the ability to neutralize singlet oxygen and scavenge radicals to inhibit lipid peroxidation, protect sperm from free radicals, and improve semen quality.

### Gene expression of *SOD1, SOD2, CAT, GPX1*, and GPX4

**Fig. 6:**
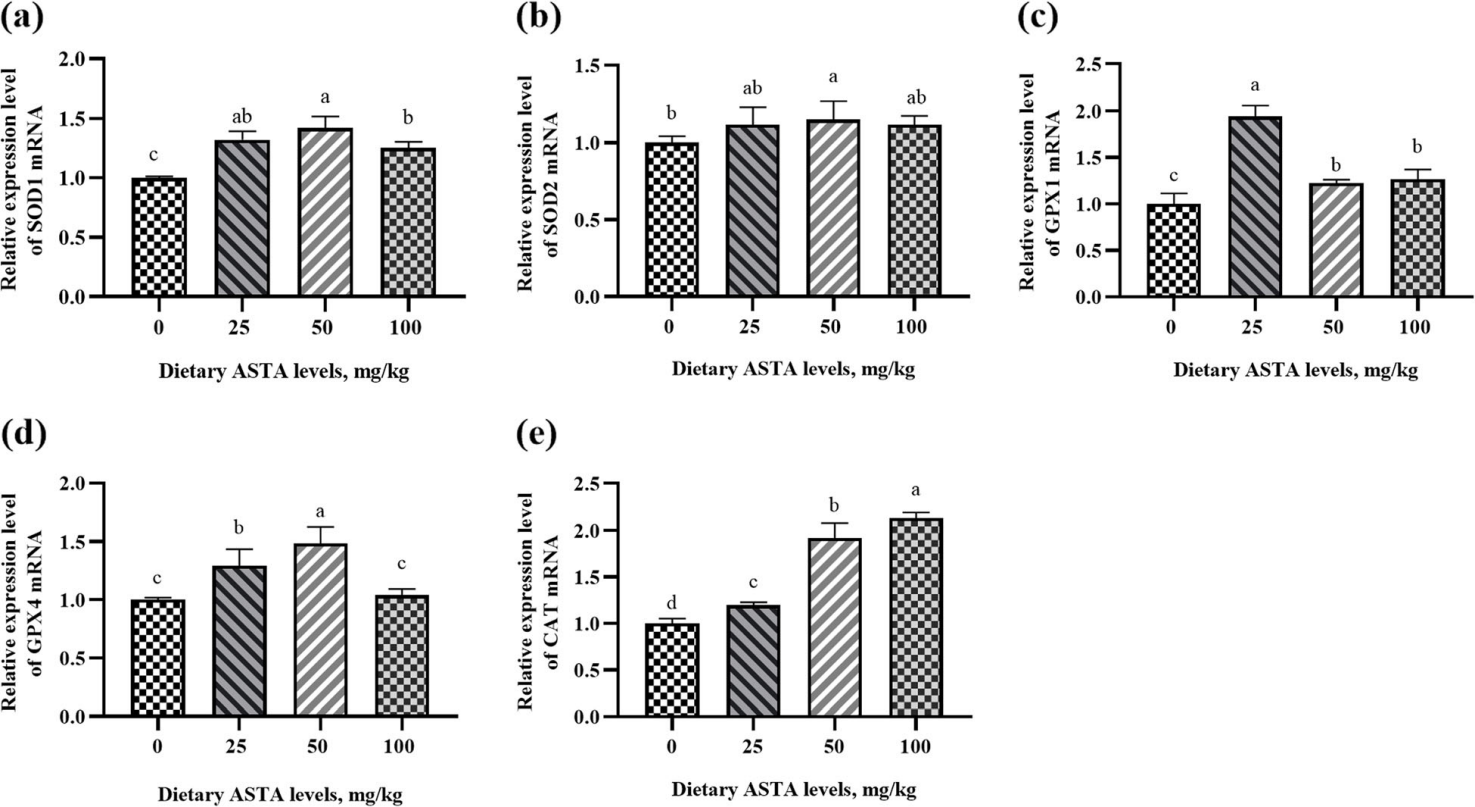
Effects of dietary natural astaxanthin (ASTA) supplementation on the mRNA expression of antioxidant enzymes, including Cu-Zn superoxide dismutase (*SOD1*) **(a)**, Mn superoxide dismutase (*SOD2*) **(b)**, glutathione peroxidase 1 (*GPX1*) **(c)**, peroxidase 4 (*GPX4*) **(d)**, and catalase (*CAT*) **(e)**, relative to that of β-actin (*ACTB*). The values are expressed as the mean ± standard deviation of six birds per treatment group. ^a–d^ Means within a row with no common superscripts differ significantly (*P* < 0.05)

The activity of an antioxidant enzyme is closely related to its gene expression [45]. The mRNA levels of *SOD1*, *SOD2*, *CAT*, *GPX1*, and *GPX4* were determined to evaluate the effects of ASTA on the gene expression of antioxidant enzymes in aging rooster testis tissues (Fig. 6). In the present study, the *SOD1* and *SOD2* mRNA levels in the 50 mg/kg ASTA group were higher than those in the other groups (*P* < 0.05). SOD1 is mainly found in the cytoplasm while SOD2 is found in the mitochondria, and ASTA has both lipophilic and hydrophilic properties, which can directly produce effects in or outside the cell membrane [46]. This may be the reason the gene expression of *SOD1* and *SOD2* was improved by adding dietary ASTA. Additionally, in the dietary ASTA supplementation group, the *GPX1* and *GPX4* mRNA levels were significantly increased relative to those in the control group (*P* < 0.05). However, there were no differences in the *GPX1* mRNA levels among the 50 and 100 mg/kg ASTA-treated groups. Furthermore, an increase in dietary ASTA supplementation from 0 to 100 mg/kg linearly increased the mRNA expression of *CAT* (*P* < 0.05). Similarly, in a previous study, dietary ASTA increased the oxidative damage repair potential of mice and upregulated the mRNA levels of *GPX1*, *SOD1*, *SOD2*, and *CAT* in the liver and kidneys [47]. Therefore, it would be interesting to further study the mechanism of how dietary ASTA affects semen quality in aging roosters by improving the antioxidant defense system.

### Gene and protein expression in the MAPK/Nrf2 pathway

**Fig. 7:**
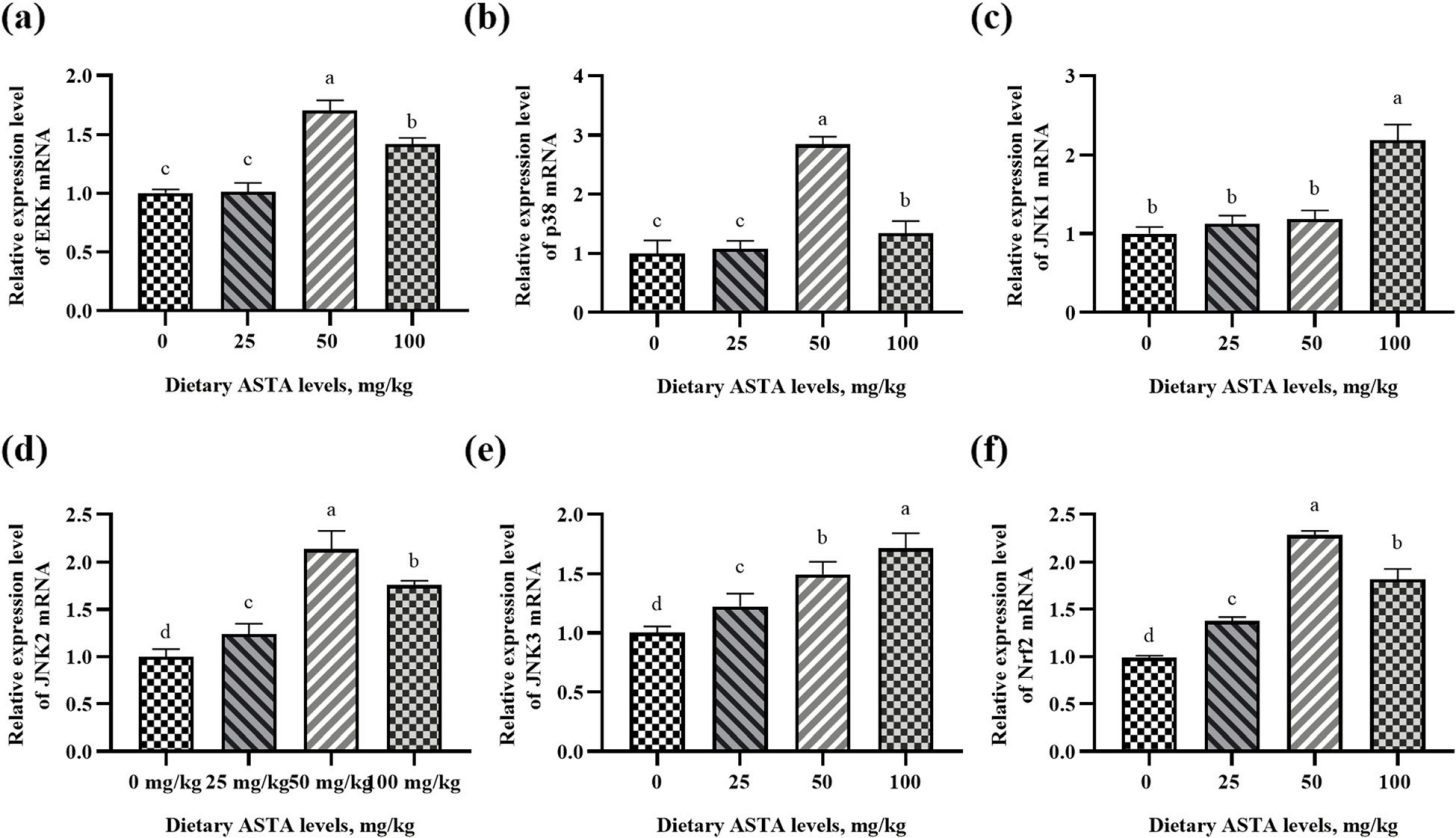
Effects of dietary natural astaxanthin (ASTA) supplementation on the mRNA expression of the mitogen-activated protein kinase/nuclear factor-erythroid 2-related factor 2 (MAPK/Nrf2) signaling pathway, including extracellular signal-regulated kinase (*ERK*) **(a)**, *p38* **(b)**, c-Jun N-terminal kinase 1 (*JNK1*) **(c)**, c-Jun N-terminal kinase 2 (*JNK2*) (**d**), c-Jun N-terminal kinase 3 (*JNK3*) **(e)**, and *Nrf2* **(f)** relative to that of β-actin (*ACTB*). The values are expressed as the mean ± standard deviation of six birds per treatment group. ^a–d^ Means within a row with no common superscripts differ significantly (*P* < 0.05)

**Fig. 8:**
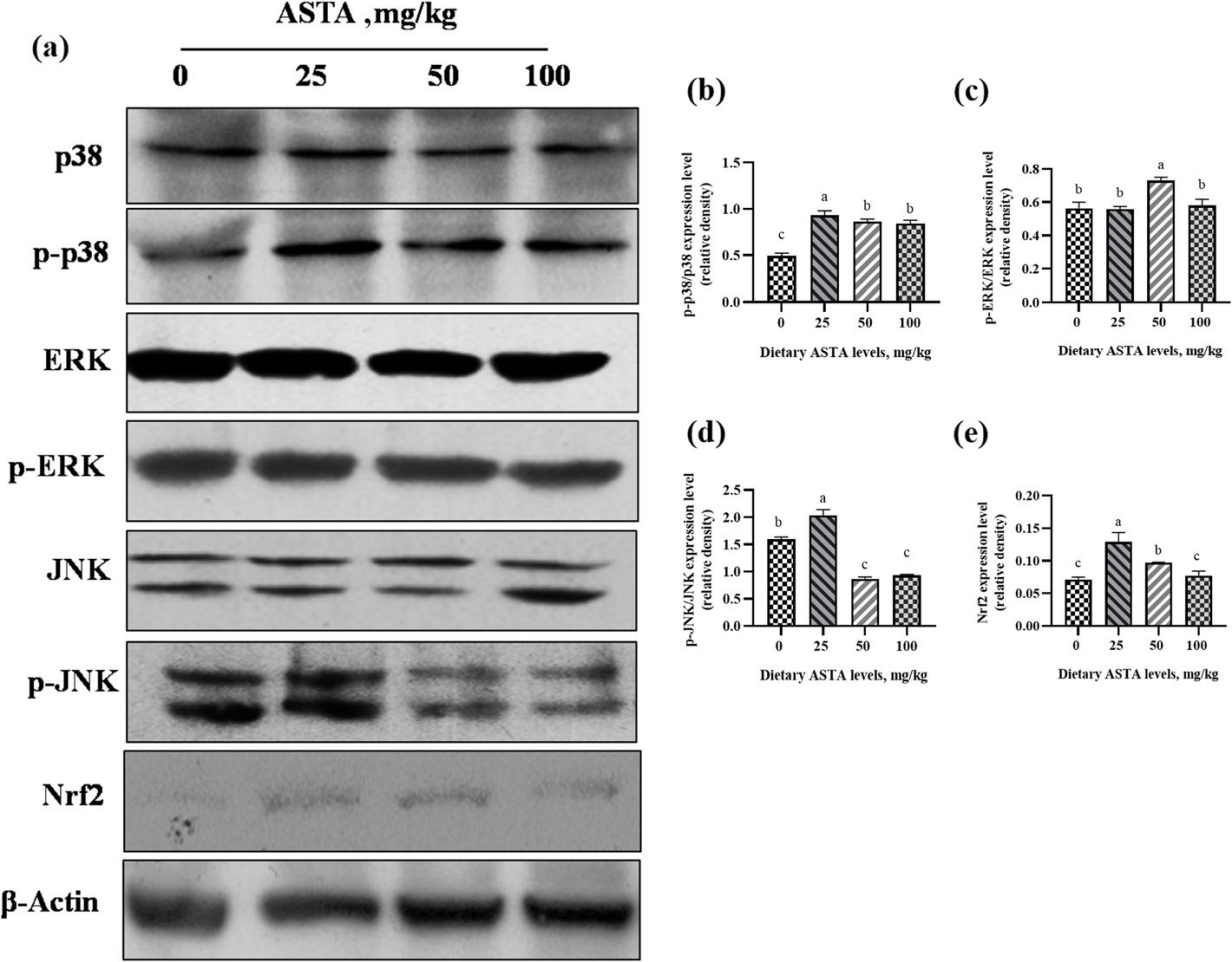
Effects of dietary natural astaxanthin (ASTA) on the expression of proteins related to the mitogen-activated protein kinase/nuclear factor-erythroid 2-related factor 2 (MAPK/Nrf2) pathway in the testis tissues of aging layer breeder roosters. The experimental groups included the control group (0 mg/kg) and ASTA groups (25–100 mg/kg). The results are expressed as the mean ± standard deviation (*n* = 3). ^a–c^ Means within a row with no common superscripts differ significantly (*P* < 0.05)

Nrf2 plays an important role in the antioxidant response, and it has been reported that the MAPK kinase pathway regulates the Nrf2 action. When the nuclear transcription factor Nrf2 is phosphorylated and activated by MAPKs (such as ERK and p38), it can be translocated to the nucleus, leading to the upregulation of antioxidant enzyme expression [22]. Therefore, to clarify whether ASTA could activate this pathway, the gene and protein expression in the MAPK/Nrf2 pathway in testis tissues were measured in the four groups, and the results are presented in Figs. 7 and 8. In this study, significant differences in the mRNA levels of *Nrf2*, *ERK*, *p38*, and *JNK* were observed among all ASTA-treated groups in the testes (Fig. 7). Briefly, when the concentration of dietary ASTA reached 50 mg/kg, the mRNA levels of *Nrf2*, *ERK*, *p38*, and *JNK2* were significantly higher than those in the control group (*P* < 0.05) and reached the maximum. A significant increase in *JNK1* mRNA expression was observed in the 100 mg/kg ASTA group (*P* < 0.05). Moreover, an increase in dietary ASTA from 0 to 100 mg/kg linearly enhanced the mRNA expression of *JNK3* (*P* < 0.05). This finding indicated that dietary ASTA increased the gene expression of *Nrf2* and upregulated the expression of upstream signals (MAPKs). Western blot analysis revealed that dietary ASTA effectively elevated (*P* < 0.05) the ratio of the phosphorylated MAPKs (p38, ERK, and JNK) to total MAPKs (p38, ERK, and JNK) compared to that in the control (Fig. 8). Briefly, the results showed that ASTA treatment remarkably upregulated the p-p38, p-ERK, and p-JNK expression in the testes (*P* < 0.05). In particular, the p-p38, p-JNK, and Nrf2 protein levels in the 25 mg/kg ASTA group were higher than those in the other groups. In addition, when the concentration of dietary ASTA reached 50 mg/kg, p-ERK protein levels were significantly higher than those in the control group (*P* < 0.05) and reached the maximum. However, Niu et al. [48] observed an opposite result that dietary ASTA supplementation did not affect the p-38 and p-JNK levels but significantly increased the p-ERK levels. Differences in test subjects, and test conditions may explain these discrepant results.

### Relationships among the MAPK/Nrf2 pathway, antioxidant enzymes, and semen quality

Aging is a complex phenomenon that is associated with an irreversible and progressive decline in body function due to biochemical and morphological changes [49]. In the process of aging, there are obvious characteristics such as increase in oxidative stress, a decline in cell function, and defects in tissues and organs [50]. In aging male animals, morphological changes in the testes include a reduction in the volume and quantity of germ cells, which leads to a decrease in sperm quality and fertilization [51]. Therefore, in this study, we explored whether dietary ASTA can improve the semen quality of aging roosters by activating the MAPK/Nrf2 pathway to enhance the service life of aging roosters. The MAPK signaling pathway is involved in many cellular functions, such as inflammation, cell proliferation, cell differentiation, and cell death [18]. Furthermore, the MAPK signaling pathway can regulate the expression of antioxidant enzymes in various cell types and adapt to various extracellular stresses through the Nrf2/ARE axis [52]. Nrf2 is a major factor that regulates antioxidant responses, and dissociates from Keap1 upon the oxidative response and translocates to the nucleus, wherein it is phosphorylated at serine 40 by the MAPK pathway, leading to the upregulation of the gene expression of antioxidant enzymes [53]. There is a high proportion of polyunsaturated fatty acids in the plasma membrane of bird sperm, and with an increase in age, the antioxidant capacity of sperm decreases. Once the plasma membrane of bird sperm is exposed to ROS, it is prone to lipid peroxidation [54]. Thus, the decrease in total antioxidant capacity of semen is one of the reasons for the decline of fertilization ability in aging roosters [55]. In the current study, our results revealed that dietary ASTA elevated the MAPK phosphorylation (p38, ERK, JNK), and the mRNA and protein expression of Nrf2 were remarkably enhanced by adding 50 mg/kg ASTA in the diets, which was in agreement with a previous study [56]. In addition, the *SOD1*, *SOD2*, *CAT*, *GPX1*, and *GPX4* mRNA levels were higher in the dietary ASTA group than those in the control group, which led to the enhancement of antioxidant capacity and semen quality in aging roosters. These results indicate that dietary ASTA can activate the MAPK/Nrf2 pathway, upregulate Nrf2 transcription and translation, and promote the expression of downstream antioxidant enzyme genes, enhancing the antioxidant capacity and improving the semen quality in aging roosters.

## Conclusions

**Fig. 9:**
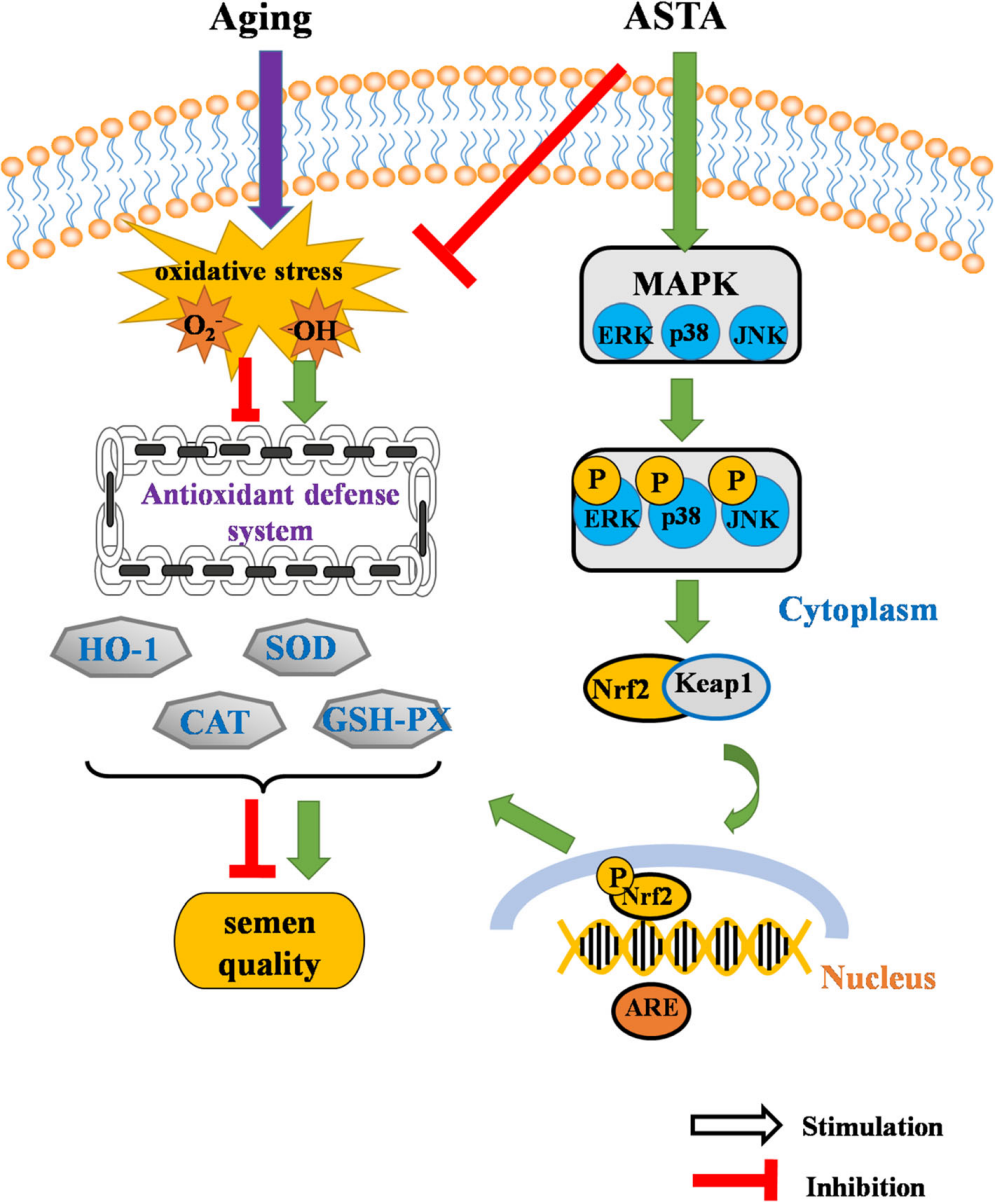
Schematic diagram summarizing the mechanisms by which natural astaxanthin (ASTA) promotes the antioxidant defense system in aging roosters. The antioxidant defense system is down regulated in the natural aging process in roosters. ASTA attenuated the oxidative stress in the testes via the activation of the mitogen-activated protein kinase/nuclear factor-erythroid 2-related factor 2 (MAPK/Nrf2) pathway to attenuate the age-related sub-fertility in aging layer breeder roosters. ARE, antioxidant responsive element; CAT, catalase; GSH-Px, glutathione peroxidase; ERK, extracellular signal-related kinase; JNK, c-Jun N-terminal kinase; SOD, superoxide dismutase

In summary, the results of this study confirmed our hypothesis that dietary ASTA supplementation improves the semen quality of aging roosters, as reflected by the upregulation of the antioxidant system (Fig. 9). Therefore, our findings suggest that dietary ASTA could attenuate age-related sub-fertility in aging layer breeder roosters. However, the possible mechanisms by which ASTA ameliorates sperm quality are not well understood. Therefore, it is necessary to evaluate the effect of dietary ASTA on semen quality via the MAPK/Nrf2 signaling pathway *in vitro*.

## Data Availability

The data for the current study are available from the corresponding author upon reasonable request.

## Abbreviations

*ACTB*: β-actin
ALH: Amplitude of lateral head displacement
ARE: Antioxidant responsive element
ASTA: Aatural astaxanthin
BCF: Beat cross frequency
CAT: Catalase
GPX1: Glutathione peroxidase 1
GPX4: Glutathione peroxidase 4
GSH-Px: Glutathione peroxidase
ERK: Extracellular signal-regulated kinase
JNK1: c-Jun N-terminal kinase 1
JNK2: c-Jun N-terminal kinase 2
JNK3: c-Jun N-terminal kinase 3
LIN: Linearity
MAPK: Mitogen-activated protein kinase
MDA: Malondialdehyde
Nrf2: Nuclear factor-erythroid 2-related factor 2
SOD: Superoxide dismutase
SOD1: Cu-Zn superoxide dismutase
SOD2: Mn superoxide dismutase
STR: Straightness
T-AOC: Total antioxidant capacity
VAP: Average path velocity
VCL: Curvilinear velocity
VSL: Straight linear velocity
WOB: Wobble of the curvilinear trajectory
